# T-RFPred: a nucleotide sequence size prediction tool for microbial community description based on terminal-restriction fragment length polymorphism chromatograms

**DOI:** 10.1186/1471-2180-10-262

**Published:** 2010-10-15

**Authors:** Antonio Fernàndez-Guerra, Alison Buchan, Xiaozhen Mou, Emilio O Casamayor, José M González

**Affiliations:** 1 Department of Continental Ecology-Biogeodynamics & Biodiversity Interactions, Centre d'Estudis Avançats de Blanes, CSIC, E-17300 Blanes, Spain; 2 Department of Microbiology, University of Tennessee, Knoxville, TN 37914, USA; 3 Department of Biological Sciences, Kent State University, Kent, OH 44242, USA; 4 Department of Microbiology, University of La Laguna, E-38206 La Laguna, Spain

## Abstract

**Background:**

Terminal-Restriction Fragment Length Polymorphism (T-RFLP) is a technique used to analyze complex microbial communities. It allows for the quantification of unique or numerically dominant phylotypes in amplicon pools and it has been used primarily for comparisons between different communities. T-RFPred, Terminal-Restriction Fragment Prediction, was developed to identify and assign taxonomic information to chromatogram peaks of a T-RFLP fingerprint for a more comprehensive description of microbial communities. The program estimates the expected fragment size of representative 16S rRNA gene sequences (either from a complementary clone library or from public databases) for a given primer and restriction enzyme(s) and provides candidate taxonomic assignments.

**Results:**

To show the accuracy of the program, T-RFLP profiles of a marine bacterial community were described using artificial bacterioplankton clone libraries of sequences obtained from public databases. For all valid chromatogram peaks, a phylogenetic group could be assigned.

**Conclusions:**

T-RFPred offers enhanced functionality of T-RFLP profile analysis over current available programs. In particular, it circumvents the need for full-length 16S rRNA gene sequences during taxonomic assignments of T-RF peaks. Thus, large 16S rRNA gene datasets from environmental studies, including metagenomes, or public databases can be used as the reference set. Furthermore, T-RFPred is useful in experimental design for the selection of primers as well as the type and number of restriction enzymes that will yield informative chromatograms from natural microbial communities.

## Background

Terminal-Restriction Fragment Length Polymorphism (T-RFLP) analysis of 16S rRNA gene amplicons is a rapid fingerprinting method for characterization of microbial communities [1,2]. It is based on the restriction endonuclease digestion profile of fluorescently end-labeled PCR products. The digested products are separated by capillary gel electrophoresis, detected and registered on an automated sequence analyzer. Each T-RF is represented by a peak in the output chromatogram and corresponds to members of the community that share a given terminal fragment size. Peak area is proportional to the abundance of the T-RF in the PCR amplicon pool, which can be used as a proxy for relative abundance in natural populations [3]. This method is rapid, relatively inexpensive and provides distinct profiles that reflect the taxonomic composition of sampled communities. Although it has extensively been used for comparative purposes, a T-RFLP fingerprint alone does not allow for conclusive taxonomic identification of individual phylotypes because it is technically challenging to recover terminal fragments for direct sequencing. However, when coupled with sequence data for representative 16S rRNA genes, T-RF identification is feasible (e.g. [4-6]). Here we describe a method to assign the T-RF peaks generated by T-RFLP analysis with either 16S rRNA gene sequences obtained from clone libraries of the same samples, metagenome sequences or data from public 16S rRNA sequence databases. T-RFPred can thus be used to classify T-RFs from T-RFLP profiles for which reference clone libraries are not available, albeit with lower phylogenetic resolution, by taking advantage of the wealth of 16S rRNA gene sequence data available from metagenome studies and public databases such as the Ribosomal Database Project (RDP) [7] or SILVA [8]. Metagenome sequencing studies from a variety of environments are accumulating at a rapid pace. While most often partial gene sequences, these libraries have the advantage that they are less subject to biases of other PCR-based techniques (see e. g. [9] for a review) and, thus, can better represent the original community structure. Furthermore, both metagenome and pyrosequencing of tagged 16S rRNA gene amplicons provides unprecedented coverage of 16S rRNA gene diversity in specific environments. Therefore, these types of datasets are valuable references when attempting to taxonomically classify T-RF peaks from diverse microbial communities.

Tools have been previously developed to perform in silico digestions of 16S rRNA gene sequences and/or to assign a taxonomic label to the chromatograms. Such programs include TAP-TRFLP [10], MiCA [11], T-RFLP Phylogenetic Assignment Tool (PAT; [12]), TReFID [13], TRAMPR [14], an ARB-software integrated tool [15] and TRiFLe [16]. Table 1 contains some of the essential features of these packages. The most obvious advantage of T-RFPred as compared with other available software applications is that the program handles either partial or full-length user input sequences. This is because T-RFPred retrieves complete sequences of close relatives from the public databases for T-RF assignments and at the same time it taxonomically bins the clone sequences. Furthermore, it can use large sequence datasets of virtually any size as reference sets in taxonomic assignments. T-RFPred is exclusive to 16S rRNA gene sequences and designed to exploit the full potential of T-RFLP profiles and their use in the description of prokaryotic communities.

**Table 1:** Characteristics of the available software to assign a phylogenetic label to the chromatogram fragment peaks

Software package	Characteristics	Reference
TAP-TRFLP	Web-based. Although it can be accessed through the older version of the Ribosomal Database Project, it has not been updated.	[10]
MiCA	Web-based. Newest version (MiCA 3) allows the selection of primers and in silico digestion of database sequences. Does not allow for user input sequences.	[11]
T-RFLP Phylogenetic Assignment Tool (PAT)	Web-based. Contains database of terminal restriction fragment sizes. Allows for the upload of fragment size database.	[12]
TReFID	Downloadable. Databases include 16S rRNA gene, dinitrogenase reductase gene (*nifH*) and nitrous oxide reductase gene (*nosZ*). Limited number of sequences although the user could expand it.	[13]
TRAMPR	R package. Based on a database of known T-RFLP profiles that can be constructed by the user. Loads data directly from ABI output files. Allows analysis with any type of gene, primer set and restriction enzyme.	[14]
ARB-software integrated tool (TRF-CUT)	Part of the ARB software. Allows for user input sequences that need to be aligned before analysis. Any type of gene could be analyzed.	[15]
TRiFLe	Java based. Allows for user input sequences. Can analyze any type of gene.	[16]
T-RFPred	Handles large database, such as 16S rRNA sequences from metagenomes, of user input clone sequences that do not need to be full length; multiple platforms. Makes use of the Ribosomal Database Project sequence database, which updates regularly. User needs to install Perl, Bioperl, BLAST and EMBOSS.	This study

Complete sequence at least at the 5'-end of the sample sequence is needed in every case except for T-RFPred, as this program finds the closest related sequence in the Ribosomal Database Project database by BLASTN.

## Implementation

T-RFPred is coded in Perl and uses the BioPerl Toolkit [17], *fuzznuc *from the EMBOSS package [18] and the BLASTN program from the NCBI BLAST suite [19]. T-RFPred has been tested in Unix-like environments, but runs in all the operating systems able to execute Perl, BioPerl, BLAST and EMBOSS; a ready-to-use VMware virtual image is also available for download at http://nodens.ceab.csic.es/t-rfpred/.

An interactive shell guides the user through the multiple steps of the analysis. Users can choose to analyze archaeal or bacterial sequences using either forward or reverse primers. The primer search utilizes *fuzznuc*, which allows the user to select the number of nucleotide ambiguities. The program extracts a subset of sequences from the RDP database that will supplement sequence analysis of clone libraries. T-RFPred generates and exports in a tab delimited text file: (1) the fragment length for the RDP sequence with the best BLASTN hit to the input sequence(s), (2) the estimated fragment length for the input sequence, (3) the gap length for the input sequence, (4) the percent identity between the input sequence and the best hit RDP sequence and (5) the taxonomic classification. The BLASTN search results and the Smith-Waterman alignments [20] are saved to allow the user to manually check the results.

### Database

The program uses a custom version of the aligned RDP as a flat file in FASTA format, where the header has been modified to include the NCBI taxonomic information and the forward/reverse position of the first non-gap character from the RDP alignment. T-RFPred exploits the Bio::DB::Flat capabilities from BioPerl to index the RDP flat file for the rapid retrieval of 16S rRNA gene sequences. All restriction enzymes available in REBase [21] are stored in a flat file and available for use in the analysis. A list of frequently used forward and reverse primers is available, although the user may also input custom primers.

### Algorithm

**Figure 1:**
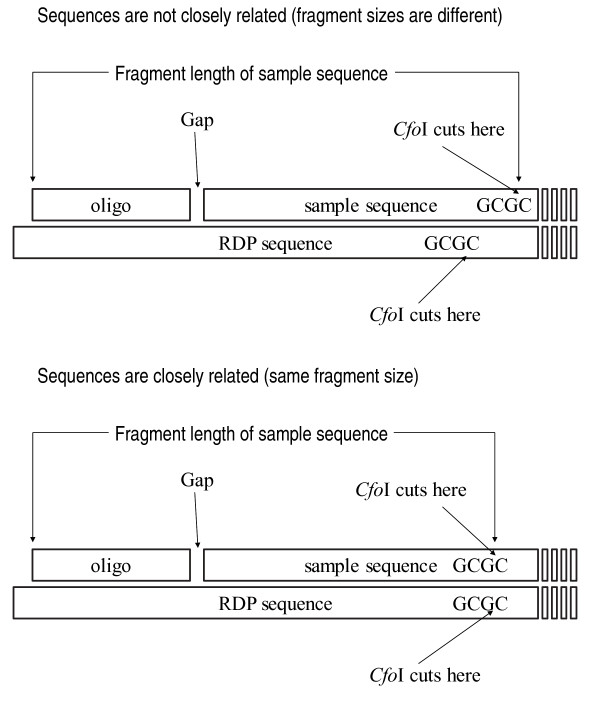
**Description of the method to estimate the length of the terminal-fragment size for partial 16S rRNA sequences**. The closest sequences (by homology search) in the RDP database are used to estimate the length of the fragment and its phylogenetic affiliation. The primer sequence is fluorescently labeled and it is close to the 5' end of the 16S rDNA gene. 'Gap' is the missing part of the sequence between the position of the primer and the beginning of the sequence. The position of the target sequence determines the size of the terminal fragment.

In part, the rationale for the described method was to circumvent the need for full-length 16S rRNA gene sequences from representative clone libraries. In addition to requiring multiple sequencing reactions, obtaining full-length sequences is generally complicated by the ambiguous nature of the 5' end of a sequence generated by the Sanger approach (i.e. the first 10-30 bp of a sequence are missing). When the same primer set used to generate T-RFLP profiles is also used to generate amplicons for libraries and directional sequencing of representative clones, as is often the case, in silico predictions of expected peak sizes are cumbersome. Additionally, the size of the fragment is subject to experimental error [22,23], which complicates the assignment of chromatogram peaks to specific phylogenetic groups. T-RFPred takes advantage of the most comprehensive database of 16S rRNA gene sequences (the RDP) to identify the closest related sequences for analysis to provide more definitive phylogenetic assignments of chromatogram peaks. Collectively, the Perl scripts achieve the following steps:

1. Create a subset of all the sequences in the RDP with nucleotide information spanning the region targeted by the fluorescently labeled primer and with a length > 1200 nucleotides for Bacteria and > 900 nucleotides for Archaea.

2. Convert the subset created in Step 1 into a BLAST-ready database using formatdb. Conduct a BLASTN search with the sample sequences (FASTA format) against the RDP database and extract the best hits.

3. Determine if sample sequences have the denoted restriction enzyme recognition site. If the cut site is present, proceed to Step 4. If the cut site is not present, estimate the expected fragment size using the closest RDP sequence and proceed to Step 5.

4. Generate a Smith-Waterman alignment of the sample sequence with the best hit from the RDP. This will provide accurate percent identities and the start/end positions of the alignment needed to estimate the fragment sizes.

5. Obtain the position of the restriction enzyme recognition site in the aligned sample sequence and the primer position in the RDP sequence. Use the RDP sequence to calculate the number of nucleotides in the gap between the primer and the start position of the Smith-Waterman alignment as shown in Figure 1.

6. Assign a taxonomic classification using the best RDP BLAST hit.

## Results and Discussion

We have developed a computational method to provide putative phylogenetic affinities of chromatogram peaks of 16S rRNA gene T-RFLP profiles. Additional file 1, Supplementary Tables S1-S3 show the typical output of T-RFPred for the clone sequences from González et al. [4], Mou et al. [5], and Pinhassi et al. [6], respectively. The T-RFPred output provides the estimated fragment size of the digested clone sequences as well as a user defined number of closest relatives. This feature is valuable for estimating the conservation of the digested product size for a given enzyme and taxonomic group analyzed.

**Figure 2:**
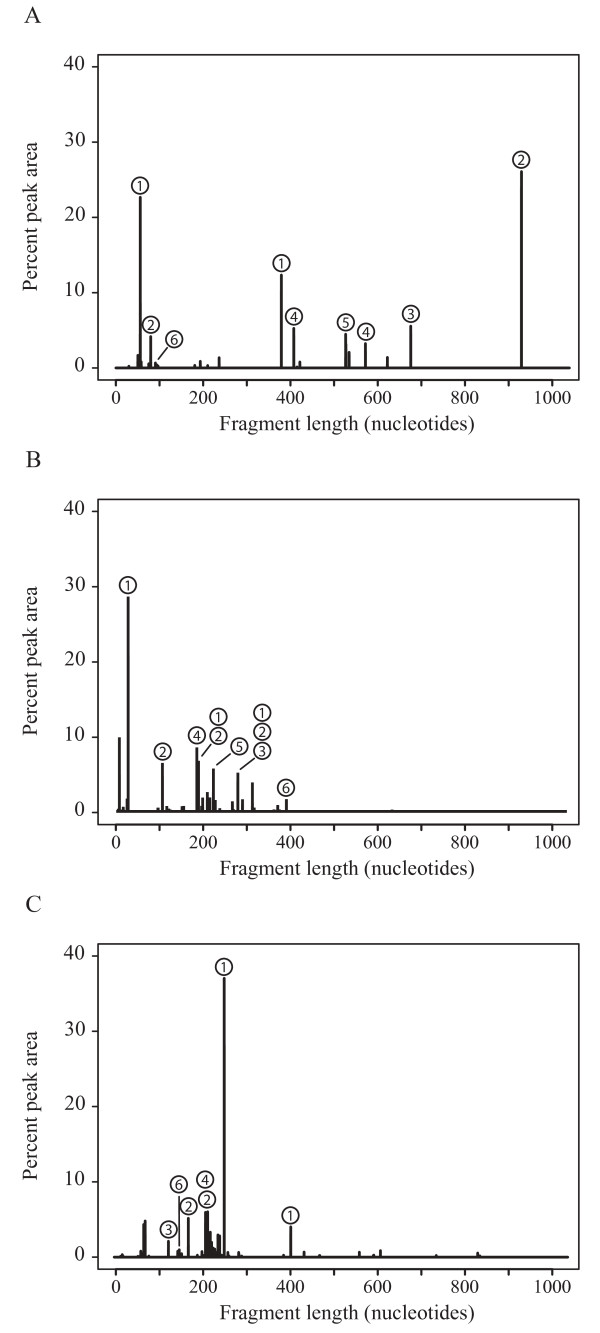
**Evaluation of the T-RFPred prediction tool**. Graphics of terminal fragment profiles generated from (A) *Cfo*I, (B) *Hae*III, and (C) *Alu*I restriction enzymes digestions of 16S rDNAs amplified from total community DNA as described in González et al. [4]. The taxonomic affiliations for the numerical labels are as follows: 1, *Roseobacter*; 2, SAR11; 3, Cyanobacteria; 4, SAR86; 5, SAR116; and 6, SAR324.

T-RFPred was also evaluated by reanalyzing chromatogram peaks from T-RFLP profiles of marine communities described in González et al. [4]. Two 16S rRNA datasets constructed from sequences from public databases, designated "4926" (4926 bacterioplankton Genbank sequences) and "GOS" (6370 Global Ocean Sampling Expedition Microbial Metagenome sequences; [24]), were analyzed with T-RFPred using three restriction enzymes (i.e., *Cfo*I, *Hae*III, and *Alu*I). Details on experimental procedure are described in the Additional File 1. The two datasets and their predicted fragment sizes and phylogenetic affiliations were used to taxonomically label the chromatogram peaks from natural samples (Figure 2). With very few exceptions, all valid fragment peaks were properly identified and in good agreement with the phylogenetic assignments reported in the literature using complementary clone libraries (Table 2). For instance, from the 4926 sequence dataset analyzed with three restriction enzymes, 124 clones yielded in silico digested fragment sizes matching peaks labeled as "1" (previously identified as alphaproteobacteria of the *Roseobacter *clade) in Figure 2. Of these clones, 90% (111 clones) were properly classified as *Roseobacter*-related, seven were Alphaproteobacteria outside the *Roseobacter *group, four Gammaproteobacteria, and two were Betaproteobacteria (Table 2). Thus, these T-RFs were labeled as *Roseobacter*. Those peaks labeled with a "2" (Figure 2) were mapped to members of the SAR11 group as 119 of the 148 sequences (80%) were from this lineage (Table 2). The chromatogram peak assignments were less ambiguous when the GOS dataset was used as the reference. With regards to T-RFs labeled 1 and 2 in Figure 2, 95% of the sequences belonged to the *Roseobacter *group and all (n = 269) sequences belonged to the SAR11 group (Table 2). Therefore, the GOS dataset was more representative of the diversity of the bacterioplankton in the natural samples. This might be because that dataset was comprised of sequences exclusively from surface seawater samples; the T-RFLP profiles analyzed were also generated from surface seawater.

**Table 2:** Phylogenetic information for the 16S rRNA sequences present in the 4926 and GOS datasets that matched selected chromatogram peaks shown in Figure 2

Dataset	Peak	Chromatograms	Number of sequences	Taxonomic group
4926	1	*Cfo*I, *Hae*III	243	Total
			146	*Roseobacter*
			53	Gammaproteobacteria
			36	Alphaproteobacteria
			4	Planctomycetes
			2	Betaproteobacteria
			1	Cyanobacteria
			1	Firmicutes
				
4926	1	*Cfo*I, *Hae*III, *Alu*I	124	Total
			111	*Roseobacter*
			7	Alphaproteobacteria
			4	Gammaproteobacteria
			2	Betaproteobacteria
				
4926	2	*Cfo*I, *Hae*III	207	Total
			152	SAR11
			51	Firmicutes
			1	Alphaproteobacteria
			1	Unclassified Bacteria
				
4926	2	*Cfo*I, *Hae*III, *Alu*I	148	Total
			119	SAR11
			29	Firmicutes
				
				
GOS	1	*Cfo*I, *Hae*III	263	Total
			231	*Roseobacter*
			18	Alphaproteobacteria
			13	Gammaproteobacteria
			1	Actinobacteria
				
GOS	1	*Cfo*I, *Hae*III, *Alu*I	243	Total
			229	*Roseobacter*
			12	Alphaproteobacteria
			1	Gammaproteobacteria
			1	Actinobacteria
				
GOS	2	*Cfo*I, *Hae*III	560	Total
			559	SAR11
			1	Alphaproteobacteria
				
GOS	2	*Cfo*I, *Hae*III, *Alu*I	269	Total
			269	SAR11

Sequences that matched the fragment sizes were analyzed using 2-3 different restriction enzymes as indicated. Alphaproteobaceria *sensu latu *refers to any bacterial sequences in the class that were not either *Roseobacter *or SAR11. See Experimental Procedures in the Additional File 1 for details.

## Conclusions

T-RFLP is a popular method for analysis of microbial communities and in silico automated methods are needed to facilitate the taxonomic identification of T-RFs in community profiles. Traditionally, computational methods to analyze T-RFLP experiments follow one of two approaches: (a) in silico simulation of the digestion of reference sequences from databases to find the most suitable enzymes that describes the microbial community organization or (b) T-RF from experiments can be binned to the in silico generated fragments to identify the taxonomic groups present in the sample. T-RFPred is designed to provide a list of candidate taxa that corresponds to the chromatogram peaks using a complementary reference clone library or public databases. Depending upon the restriction enzyme used, broad phylogenetic groups can sometimes give the same fragment size. Thus, we also determined that community profiles generated with at least two different restriction enzymes are needed for the most robust taxonomic identifications (Table 2). The method has also its caveats as is not meant to positively identify phylogenetic groups or species based upon terminal fragment length, particularly, as the identification of the sequences cannot be solely determined based on the closest BLASTN hit alone. Manual inspection of the BLASTN hits and additional efforts may also be needed for more conclusive taxonomic assignments. In the example above, we conducted homology searches (BLASTN) to a set of reference sequences from representative taxa as well as phylogenetic treeing methods to confirm the taxonomic affiliations of the GOS and 4926 sequences whose predicted fragment sizes matched a chromatogram peaks (data not shown). Despite these caveats, the position of restriction enzyme recognition sites within the 16S rDNA molecule does reflect a level of phylogeny and can be used to help guide experimental design (i.e. which and how many restriction enzymes are most appropriate for a given community) so that the most reliable results for the T-RFLP characterization of a given prokaryotic assemblage can be obtained.

In summary, T-RFPred offers an alternative, freeware and open source program for researchers using T-RFLP to examine microbial populations. The program can help researchers determine the most appropriate restriction enzyme(s) to use when designing experiments to assess community structure using the T-RFLP method. It can also provide information on the taxonomic assignments of specific T-RFs without the need for comprehensive complementary clone libraries.

## Availability and requirements

Project name: T-RFPred

Project home page: http://nodens.ceab.csic.es/t-rfpred/

Operating systems: Linux (tested in Debian, Ubuntu and RHEL), Mac OS X (tested in MacOS X 10.5 and Mac OS X 10.6), Windows (via a Xubuntu VMware image)

Programming language: Perl

Other requirements: BioPerl, BLAST and EMBOSS

License: none

Any restrictions to use by non-academics: none

## Authors' contributions

AFG wrote the script and participated in the analysis and drafting of the manuscript. XM participated in the analysis and AB in the analysis and drafting of the manuscript. EOC coordinated the study, as well as participated in writing the manuscript. JMG conceived the study, and participated in its design and coordination. JMG was also involved in the analysis and interpretation of results and drafting of the manuscript. All authors read and approved the final manuscript.

## Supplementary Material

Additional file 1 **"Project website", "Additional Experimental Procedure" and "Supplementary Tables S1-S3"**. Project website. Webpage to download T-RFPred. Additional Experimental Procedure. Origin of chromatograms and reference datasets to label the peaks on Figure 2. Supplementary Tables S1-S3. Typical output of T-RFPred for the clone sequences from [4-6], respectively. Click here for file
