# Supplementary material for: T-RFPred: a nucleotide sequence size prediction tool for microbial community description based on terminal-restriction fragment length polymorphism chromatograms
Source: BMC Microbiol. 2010 Oct 15;10:262. doi: 10.1186/1471-2180-10-262 (PMC2965158; doi:10.1186/1471-2180-10-262)
Supplement: Additional file 1 — "Project website", "Additional Experimental Procedure" and "Supplementary Tables S1-S3". Project website. Webpage to download T-RFPred. Additional Experimental Procedure. Origin of chromatograms and reference datasets to label the peaks on Figure 2. Supplementary Tables S1-S3. Typical output of T-RFPred for the clone sequences from [4-6], respectively. [file 1471-2180-10-262-S1.pdf]

Additional Information

T-RFPred can be downloaded from http://sourceforge.net/projects/t-rfpred/files/

Experimental Procedures

T-RFLP chromatograms

To test the method, chromatogram peaks from marine samples were taxonomically identified using datasets of bacterioplankton 16S rRNA sequences from GenBank. The origin of the DNA samples, PCR conditions and T-RFLP analysis are described in detail in González et al. (2000). These T-RFLP profiles were generated using the primers 8F-FAM (5'-AGAGTTTGATCCTGGCTCAG; PE Corporation) and 1522R (5'-AAGGAGGTGATCCANCCRCA). The restriction enzymes used were *Alu*I, *Hae*III and *Cfo*I (Boehringer Mannheim). 16S rRNA sequences were retrieved from GenBank by searching for strings as described in Hagström et al. (2002). Since many more sequences have been submitted since that publication, these search limits did not guarantee that all recovered sequences are derived from bacterioplankton samples. Thus, a script was written to cluster sequences with the same GenBank source field and those sequences were manually inspected to remove any that were not of marine origin. Sequences < 1000 bp in length were also removed. This left a total of 4926 sequences for further analysis, hereafter referred to as the "4926 dataset". Another set of sequences consisted of the 16S rRNA sequences > 1000 bp from the Global Ocean Sampling Expedition Microbial Metagenome (6370 sequences; designated the "GOS dataset"; Rusch et al., 2007). Due to the large size of these datasets, custom scripts were written to facilitate the taxonomic identification of the chromatogram peaks.

From these two datasets, subsets of sequences were extracted that would yield a fragment peak of a certain size on the chromatogram within a 5-10 nucleotide window

and for a given restriction enzyme. This window size was chosen because of the experimental error associated to the estimation of the fragment size (see main text). These subsets were then further analyzed to determine the expected fragment sizes for the other two restriction enzymes. Sequences whose expected sizes matched the chromatogram peaks were finally used to provide a taxonomic label for the peaks. The phylogenetic placement of the sequences used to label the peaks was determined using T-RFPred, RDP Classifier (Wang et al., 2007), as well as a local BLAST to the sequences from the type strains of reference bacterial species (Yarza et al., 2008).

Supplementary Table S1. Comparison between the phylogenetic affiliations described in González et al. (2000) and the predictions obtained by T-RFPred. eFL: predicted fragment length (bp).

González et al. 2000				T-RFPred prediction			
**Clone acc. number**	**Closest hit acc. number**	**Group**	**Similarity (%)**	**Closest hit accession number**	**eFL**	**Similiarity (%)**	**RDP Taxonomy**
AF245657	U75257	SAR11	96	AB193906	906	97.6	Root;Bacteria;Proteobacteria;Alphaproteobacteria;unclassified_Alphaproteobacteria.
AF245656	U75257	SAR11	94.6	EU801410	906	96.4	Root;Bacteria;Proteobacteria;Alphaproteobacteria;Rhizobiales;unclassified_Rhizobiales.
AF245655	L10935	SAR11	93.1	AB193894	908	98.4	Root;Bacteria;Proteobacteria;Alphaproteobacteria;unclassified_Alphaproteobacteria.
AF245654	AF104473	α-Proteobacteria	91.9	FJ202874	94	95.7	Root;Bacteria;Proteobacteria;Alphaproteobacteria;Rhodospirillales;unclassified_Rhodospirillales.
AF245653	U75257	SAR11	95.1	FJ545621	908	95.7	Root;Bacteria;Proteobacteria;Alphaproteobacteria;unclassified_Alphaproteobacteria.
AF245651	U70691	SAR11	99	DQ906730	907	99.1	Root;Bacteria;Proteobacteria;Alphaproteobacteria;unclassified_Alphaproteobacteria.
AF245650	AJ227814	α-Proteobacteria	99.3	AF082799	503	99.3	Root;Bacteria;Proteobacteria;Alphaproteobacteria;Rhodobacterales;Rhodobacteraceae;Hyphomonas.

AF245649	AF141501	γ-Proteobacteria	98.9	DQ396337	1119	98.7	Root;Bacteria;Proteobacteria;Gammaproteobacteria;unclassified_Gammaproteobacteria.
AF245648	X52172	SAR11	95.1	EF573350	182	97.1	Root;Bacteria;Proteobacteria;Alphaproteobacteria;unclassified_Alphaproteobacteria.
AF245647	X82139	γ-Proteobacteria	99.8	FJ695595	364	99.5	Root;Bacteria;Proteobacteria;Gammaproteobacteria;Alteromonadales;Pseudoalteromonadaceae;Pseudoalteromonas.
AF245646	L35504	δ-Proteobacteria	89.1	DQ396207	95	95.1	Root;Bacteria;Proteobacteria;Deltaproteobacteria;Desulfobacterales;Nitrospinaceae;Nitrospina.
AF245645	EU805021	Cytophagales	97.7	EU805021	90	97.7	Root;Bacteria;Bacteroidetes;Sphingobacteria;Sphingobacteriales;Flexibacteraceae;unclassified_Flexibacteraceae.
AF245644	AF141439	γ-Proteobacteria	91.4	AY942761	226	95.2	Root;Bacteria;Proteobacteria;Gammaproteobacteria;Oceanospirillales;unclassified_Oceanospirillales.
AF245643	AF141586	γ-Proteobacteria	95.8	FJ826169	60	94.0	Root;Bacteria;Proteobacteria;Gammaproteobacteria;unclassified_Gammaproteobacteria.
AF245642	AF001650	SAR86	99.8	FJ545592	362	99.5	Root;Bacteria;Proteobacteria;Gammaproteobacteria;unclassified_Gammaproteobacteria.
AF245641	AF001638	α-Proteobacteria	100	AY627381	82	99.5	Root;Bacteria;Proteobacteria;Alphaproteobacteria;unclassified_Alphaproteobacteria.
AF245640	AF001651	SAR86	99.8	EU799743	565	99.8	Root;Bacteria;Proteobacteria;Gammaproteobacteria;unclassified_Gammaproteobacteria.
AF245639	D30791	α-Proteobacteria	91.5	FJ826369	182	98.2	Root;Bacteria;Proteobacteria;Alphaproteobacteria;unclassified_Alphaproteobacteria.

AF245638	U70684	SAR11	99.8	FJ825912	901	99.6	Root;Bacteria;Proteobacteria;unclassified_Proteobacteria.
AF245637	AJ002563	*Roseobacter*	100	EU800060	60	100	Root;Bacteria;Proteobacteria;Alphaproteobacteria;Rhodobacterales;Rhodobacteraceae;unclassified_Rhodobacteraceae.
AF245636	AF001650	SAR86	98.5	EU919818	365	99.8	Root;Bacteria;Proteobacteria;Gammaproteobacteria;unclassified_Gammaproteobacteria.
AF245635	U70680	*Roseobacter*	99.3	EU799989	59	99.6	Root;Bacteria;Proteobacteria;Alphaproteobacteria;Rhodobacterales;Rhodobacteraceae;Thalassobacter.
AF245634	U37762	*Roseobacter*	97.3	FJ545602	340	99.7	Root;Bacteria;Proteobacteria;Alphaproteobacteria;Rhodobacterales;Rhodobacteraceae;unclassified_Rhodobacteraceae.
AF245633	AF001651	SAR86	100	EU799743	564	98.9	Root;Bacteria;Proteobacteria;Gammaproteobacteria;unclassified_Gammaproteobacteria.
AF245632	AF007259	*Roseobacter*	99.8	EU819141	61	99.9	Root;Bacteria;Proteobacteria;Alphaproteobacteria;Rhodobacterales;Rhodobacteraceae;unclassified_Rhodobacteraceae.
AF245631	U37762	*Roseobacter*	96.8	FJ545602	341	100	Root;Bacteria;Proteobacteria;Alphaproteobacteria;Rhodobacterales;Rhodobacteraceae;unclassified_Rhodobacteraceae.
AF245630	AF098495	*Roseobacter*	96.1	EU805016	61	99.9	Root;Bacteria;Proteobacteria;Alphaproteobacteria;Rhodobacterales;Rhodobacteraceae;unclassified_Rhodobacteraceae.
AF245629	AF001650	SAR86	99.5	FJ545592	366	99.1	Root;Bacteria;Proteobacteria;Gammaproteobacteria;unclassified_Gammaproteobacteria.
AF245628	AF007259	*Roseobacter*	93.4	EU799430	60	99.9	Root;Bacteria;Proteobacteria;Alphaproteobacteria;Rhodobacterales;Rhodobacteraceae;unclassified_Rhodobacteraceae.

AF245627	U70693	γ-Proteobacteria	85.4	FJ825908	579	99.7	Root;Bacteria;Proteobacteria;Gammaproteobacteria;Oceanospirillales;unclassified_Oceanospirillales.
AF245626	U78944	α-Proteobacteria	98.2	FJ826374	343	99.6	Root;Bacteria;Proteobacteria;Alphaproteobacteria;Rhizobiales;unclassified_Rhizobiales.
AF245625	U37762	*Roseobacter*	96.6	EU799470	59	98.8	Root;Bacteria;Proteobacteria;Alphaproteobacteria;Rhodobacterales;Rhodobacteraceae;unclassified_Rhodobacteraceae.
AF245624	U70687	SAR11	100	FJ825912	901	100	Root;Bacteria;Proteobacteria;unclassified_Proteobacteria.
AF245623	AF141447	α-Proteobacteria	95.8	EU394547	82	94.7	Root;Bacteria;Proteobacteria;Alphaproteobacteria;unclassified_Alphaproteobacteria.
AF245622	AF001650	SAR86	100	DQ395550	366	100	Root;Bacteria;Proteobacteria;Gammaproteobacteria;unclassified_Gammaproteobacteria.
AF245621	U37762	*Roseobacter*	97	FM958453	341	99.5	Root;Bacteria;Proteobacteria;Alphaproteobacteria;Rhodobacterales;Rhodobacteraceae;unclassified_Rhodobacteraceae.
AF245620	U70684	SAR11	100	EU804903	907	98.9	Root;Bacteria;Proteobacteria;Alphaproteobacteria;unclassified_Alphaproteobacteria.
AF245619	U78944	α-Proteobacteria	99.7	EU799979	526	99.7	Root;Bacteria;Proteobacteria;Alphaproteobacteria;unclassified_Alphaproteobacteria.
AF245618	AF141574	Cyanobacteria	100	AJ289785	667	99.7	Root;Bacteria;Cyanobacteria;Cyanobacteria;Family II;GpIIa.
AF245617	U70680	*Roseobacter*	100	FJ826118	60	99.8	Root;Bacteria;Proteobacteria;Alphaproteobacteria;Rhodobacterales;Rhodobacteraceae;Thalassobacter.

AF245615	AF007259	*Roseobacter*	94.8	DQ395864	61	97.9	Root;Bacteria;Proteobacteria;Alphaproteobacteria;Rhodobacterales;Rhodobacteraceae;unclassified_Rhodobacteraceae.
AF245614	AF098495	*Roseobacter*	95.9	EU805243	61	99.7	Root;Bacteria;Proteobacteria;Alphaproteobacteria;Rhodobacterales;Rhodobacteraceae;unclassified_Rhodobacteraceae.

Supplementary Table S2. Comparison between the phylogenetic affiliations described in Pinhassi et al. (2005) and the predictions obtained by T-RFPred. eFL: predicted fragment length (bp).

Pinhassi et al. 2005				T-RFPred prediction			
**Clone accession number**	**Closest hit accession number**	**Group**	**Identity (%)**	**Closest hit accession number**	**eFL**	**Identity (%)**	**RDP Taxonomy**
AY904518	AY386339	γ-Proteobacteria	93.4	EF379800	355	94.2	Root;Bacteria;Proteobacteria;Gammaproteobacteria;unclassified_Gammaproteobacteria.
AY904517	Z88589	γ-Proteobacteria	99.1	FJ745164	367	98.7	Root;Bacteria;Proteobacteria;Gammaproteobacteria;unclassified_Gammaproteobacteria.
AY904516	AJ627987	α-Proteobacteria	90.6	FJ745066	62	98.0	Root;Bacteria;Proteobacteria;Alphaproteobacteria;unclassified_Alphaproteobacteria.
AY904515	AF141586	γ-Proteobacteria	96.4	EU919816	61	97.6	Root;Bacteria;Proteobacteria;Gammaproteobacteria;unclassified_Gammaproteobacteria.
AY904514	AY568820	γ-Proteobacteria	93.7	FJ545459	206	94.6	Root;Bacteria;Proteobacteria;Gammaproteobacteria;Alteromonadales;Incertae sedis 7;unclassified_Incertae sedis 7.
AY904513	AY580445	α-Proteobacteria	99.6	FJ745263	61	99.9	Root;Bacteria;Proteobacteria;Alphaproteobacteria;Rhodobacterales;Rhodobacteraceae;Roseovarius.
AY904512	AY033298	α-Proteobacteria	99.1	EU804360	82	99.5	Root;Bacteria;Proteobacteria;Alphaproteobacteria;unclassified_Alphaproteobacteria.
AY904511	AY580433	Planctomycetes	93.2	EU617889	1439	93.6	Root;Bacteria;Planctomycetes;Planctomycetacia;Planctomycetales;Planctomycetaceae;unclassified_Planctomycetaceae.

AY904510	AY172836	Cyanobacteria	99.5	FM995188	670	99.5	Root;Bacteria;Cyanobacteria;Cyanobacteria;Family II;GpIIa.
AY904509	Z88582	α-Proteobacteria	99.6	FJ745263	61	99.6	Root;Bacteria;Proteobacteria;Alphaproteobacteria;Rhodobacterales;Rhodobacteraceae;Roseovarius.
AY904508	AY038531	α-Proteobacteria	97.1	FJ203089	61	99.7	Root;Bacteria;Proteobacteria;Alphaproteobacteria;Rhodobacterales;Rhodobacteraceae;unclassified_Rhodobacteraceae.
AY904507	AY344412	Planctomycetes	88.2	EF575129	61	99.8	Root;Bacteria;Planctomycetes;Planctomycetacia;Planctomycetales;Planctomycetaceae;unclassified_Planctomycetaceae.
AY904506	AF473915	α-Proteobacteria	97.9	FJ202463	61	97.4	Root;Bacteria;Proteobacteria;Alphaproteobacteria;Rhodobacterales;Rhodobacteraceae;Shimia.
AY904505	AY038415	Unknown	89.3	FJ425635	91	93.6	Root;Bacteria;Proteobacteria;unclassified_Proteobacteria.
AY904504	AY580815	Unknown	96.5	FM165252	203	89.5	Root;Bacteria;Proteobacteria;Alphaproteobacteria;Rhizobiales;unclassified_Rhizobiales.
AY904503	AY136124	α-Proteobacteria	98.7	FJ403092	61	99.2	Root;Bacteria;Proteobacteria;Alphaproteobacteria;Rhodobacterales;Rhodobacteraceae;Thalassobius.
AY904502	AY345413	α-Proteobacteria	96.8	FJ202229	61	98.0	Root;Bacteria;Proteobacteria;Alphaproteobacteria;Rhodobacterales;Rhodobacteraceae;unclassified_Rhodobacteraceae.
AY904501	AY125371	Cyanobacteria	99.4	FJ937843	669	99.8	Root;Bacteria;Cyanobacteria;Cyanobacteria;Family II;GpIIa.
AY904500	Z88589	γ-Proteobacteria	99.2	FJ745164	367	99.0	Root;Bacteria;Proteobacteria;Gammaproteobacteria;unclassified_Gammaproteobacteria.
AY904499	AY697876	α-Proteobacteria	100	FJ825896	901	100	Root;Bacteria;Proteobacteria;Alphaproteobacteria;unclassified_Alphaproteobacteria.

AY904498	U13159	α-Proteobacteria	99.5	EU804543	907	100	Root;Bacteria;Proteobacteria;Alphaproteobacteria;unclassified_Alphaproteobacteria.
AY904497	AY125371	Cyanobacteria	99.6	FM995188	670	99.8	Root;Bacteria;Cyanobacteria;Cyanobacteria;Family II;GpIIa.
AY904496	Z88579	γ-Proteobacteria	98.5	EF575314	61	99.4	Root;Bacteria;Proteobacteria;Gammaproteobacteria;Oceanospirillales;Alcanivoraceae;Alcanivorax.
AY904495	AJ002565	α-Proteobacteria	99.8	AB294940	61	95.3	Root;Bacteria;Proteobacteria;Alphaproteobacteria;Rhodobacterales;Rhodobacteraceae;unclassified_Rhodobacteraceae.
AY904494	AY038571	α-Proteobacteria	99.8	EU805016	61	99.9	Root;Bacteria;Proteobacteria;Alphaproteobacteria;Rhodobacterales;Rhodobacteraceae;unclassified_Rhodobacteraceae.
AY904493	AY580483	α-Proteobacteria	99.3	EU804746	907	99.8	Root;Bacteria;Proteobacteria;Alphaproteobacteria;unclassified_Alphaproteobacteria.
AY904492	AY580474	α-Proteobacteria	99.8	EU804807	907	100	Root;Bacteria;Proteobacteria;Alphaproteobacteria;unclassified_Alphaproteobacteria.
AY904491	AY033296	Actinobacteria	100	FJ826502	39	100	Root;Bacteria;unclassified_Bacteria.
AY904490	AY580445	α-Proteobacteria	99.4	FJ745263	61	99.8	Root;Bacteria;Proteobacteria;Alphaproteobacteria;Rhodobacterales;Rhodobacteraceae;Roseovarius.
AY904489	AY125371	Cyanobacteria	99.6	FM995188	670	99.8	Root;Bacteria;Cyanobacteria;Cyanobacteria;Family II;GpIIa.
AY904488	AY499416	α-Proteobacteria	99.6	EU802885	907	100	Root;Bacteria;Proteobacteria;Alphaproteobacteria;unclassified_Alphaproteobacteria.
AY904487	AY193157	γ-Proteobacteria	91.8	FM242293	366	90.9	Root;Bacteria;Proteobacteria;Gammaproteobacteria;Oceanospirillales;unclassified_Oceanospirillales.

AY904486	AF268220	γ-Proteobacteria	98.9	EU394545	908	99.6	Root;Bacteria;Proteobacteria;Alphaproteobacteria;Rhizobiales;unclassified_Rhizobiales.
AY904485	AY033299	α-Proteobacteria	97.1	EU804104	907	99.4	Root;Bacteria;Proteobacteria;Alphaproteobacteria;unclassified_Alphaproteobacteria.
AY904484	AY093481	α-Proteobacteria	98.8	EU805335	907	99.4	Root;Bacteria;Proteobacteria;Alphaproteobacteria;unclassified_Alphaproteobacteria.
AY904483	AY172836	Cyanobacteria	99.2	FM995188	670	99.8	Root;Bacteria;Cyanobacteria;Cyanobacteria;Family II;GpIIa.
AY904482	AY172836	Cyanobacteria	99.6	FM995188	670	100	Root;Bacteria;Cyanobacteria;Cyanobacteria;Family II;GpIIa.
AY904481	AY664120	δ-Proteobacteria	97.9	FJ155032	91	99.0	Root;Bacteria;Proteobacteria;Deltaproteobacteria;unclassified_Deltaproteobacteria.
AY573531	U62913	Bacteroidetes	94.4	EU287248	96	90.9	Root;Bacteria;Bacteroidetes;Flavobacteria;Flavobacteriales;Flavobacteriaceae;Algibacter.

Supplementary Table S3. Comparison between the phylogenetic affiliations described in Mou et al. (2005) and the predictions obtained by T-RFPred. eFL: predicted fragment length (bp).

**Mou et al. 2005**				**T-RFPred prediction**			
**Clone accession number**	**Closest hit accession number**	**Group**	**Identity (%)**	**Closest hit accession number**	**eFL**	**Identity (%)**	**RDP Taxonomy**
AY476805	AF546929	β-Proteobacteria	96	EU104172	205	100	Root;Bacteria;Proteobacteria;Betaproteobacteria;Burkholderiales;Comamonadaceae;Curvibacter.
AY476804	AY609198	*Rhodoferax*	97	EF632894	206	99.0	Root;Bacteria;Proteobacteria;Betaproteobacteria;Burkholderiales;Comamonadaceae;Rhodoferax.
AY476802	AY580466	*Roseobacter*	99	FJ203648	61	98.4	Root;Bacteria;Proteobacteria;Alphaproteobacteria;Rhodobacterales;Rhodobacteraceae;unclassified_Rhodobacteraceae.
AY476801	AY580764	γ-Proteobacteria	93	EF379837	231	93.0	Root;Bacteria;Proteobacteria;Gammaproteobacteria;unclassified_Gammaproteobacteria.
AY476800	AF477832	Bacteroidetes	99	EF471578	97	99.3	Root;Bacteria;Bacteroidetes;Sphingobacteria;Sphingobacteriales;Flexibacteraceae;unclassified_Flexibacteraceae.
AY476799	AY580722	Bacteroidetes	99	EF471578	98	99.5	Root;Bacteria;Bacteroidetes;Sphingobacteria;Sphingobacteriales;Flexibacteraceae;unclassified_Flexibacteraceae.
AY476795	AJ784130	γ-Proteobacteria	94	FJ154988	370	97.2	Root;Bacteria;Proteobacteria;Gammaproteobacteria;Alteromonadales;Pseudoalteromonadaceae;Pseudoalteromonas.
AY476794	AJ391181	*Roseobacter*	97	FJ869045	513	100	Root;Bacteria;Proteobacteria;Alphaproteobacteria;Rhodobacterales;Rhodobacteraceae;unclassified_Rhodobacteraceae.

AY476785	AY461670	γ-Proteobacteria	99	FJ154989	373	99.1	Root;Bacteria;Proteobacteria;Gammaproteobacteria;Alteromonadales;Pseudoalteromonadaceae;Pseudoalteromonas.
AY476784	AF227238	γ-Proteobacteria	92	FJ457244	373	99.8	Root;Bacteria;Proteobacteria;Gammaproteobacteria;Alteromonadales;Pseudoalteromonadaceae;Pseudoalteromonas.
AY476781	AY461670	γ-Proteobacteria	98	FJ876419	373	98.1	Root;Bacteria;Proteobacteria;Gammaproteobacteria;Alteromonadales;Pseudoalteromonadaceae;Pseudoalteromonas.
AY476780	AY461670	γ-Proteobacteria	99	FJ876419	371	99.1	Root;Bacteria;Proteobacteria;Gammaproteobacteria;Alteromonadales;Pseudoalteromonadaceae;Pseudoalteromonas.
AY476779	AY580722	Bacteroidetes	99	EF471578	97	99.4	Root;Bacteria;Bacteroidetes;Sphingobacteria;Sphingobacteriales;Flexibacteraceae;unclassified_Flexibacteraceae.
AY476778	AY580367	γ-Proteobacteria	95	EU487984	324	98.1	Root;Bacteria;Proteobacteria;Gammaproteobacteria;Alteromonadales;Colwelliaceae;Thalassomonas.
AY476776	AY461670	γ-Proteobacteria	99	FJ876419	372	99.1	Root;Bacteria;Proteobacteria;Gammaproteobacteria;Alteromonadales;Pseudoalteromonadaceae;Pseudoalteromonas.
AY476775	AY580722	Bacteroidetes	99	EF471578	97	99.4	Root;Bacteria;Bacteroidetes;Sphingobacteria;Sphingobacteriales;Flexibacteraceae;unclassified_Flexibacteraceae.
AY476774	Z88582	*Roseobacter*	98	FJ745263	61	99.3	Root;Bacteria;Proteobacteria;Alphaproteobacteria;Rhodobacterales;Rhodobacteraceae;Roseovarius.
AY476771	AY580445	*Roseobacter*	99	FJ745263	61	100	Root;Bacteria;Proteobacteria;Alphaproteobacteria;Rhodobacterales;Rhodobacteraceae;Roseovarius.
AY476770	AY580445	*Roseobacter*	99	FJ745263	61	98.8	Root;Bacteria;Proteobacteria;Alphaproteobacteria;Rhodobacterales;Rhodobacteraceae;Roseovarius.
AY476769	Z88582	*Roseobacter*	99	FJ745263	61	99.3	Root;Bacteria;Proteobacteria;Alphaproteobacteria;Rhodobacterales;Rhodobacteraceae;Roseovarius.

AY476767	AY461670	γ-Proteobacteria	99	FJ154989	372	99.1	Root;Bacteria;Proteobacteria;Gammaproteobacteria;Alteromonadales;Pseudoalteromonadaceae;Pseudoalteromonas.
AY476763	Z88582	*Roseobacter*	99	FJ745263	61	99.5	Root;Bacteria;Proteobacteria;Alphaproteobacteria;Rhodobacterales;Rhodobacteraceae;Roseovarius.
AY476751	AY033296	Actinobacteria	96	EU800288	39	99.2	Root;Bacteria;unclassified_Bacteria.
AY476750	AF546929	β-Proteobacteria	95	EU104172	206	99.8	Root;Bacteria;Proteobacteria;Betaproteobacteria;Burkholderiales;Comamonadaceae;Curvibacter.
AY476741	AY345438	*Roseobacter*	98	EF574092	341	99.1	Root;Bacteria;Proteobacteria;Alphaproteobacteria;Rhodobacterales;Rhodobacteraceae;Loktanella.
AY476740	AY159887	*Sulfitobacter*	99	FJ826092	60	99.6	Root;Bacteria;Proteobacteria;Alphaproteobacteria;Rhodobacterales;Rhodobacteraceae;Sulfitobacter.
AY476739	AY580445	*Roseobacter*	100	FJ745263	61	100	Root;Bacteria;Proteobacteria;Alphaproteobacteria;Rhodobacterales;Rhodobacteraceae;Roseovarius.
AY476738	AF053734	γ-Proteobacteria	93	EF379837	213	96.6	Root;Bacteria;Proteobacteria;Gammaproteobacteria;unclassified_Gammaproteobacteria.
AF547425	AF468380	*Sulfitobacter*	97	FJ826092	58	98.9	Root;Bacteria;Proteobacteria;Alphaproteobacteria;Rhodobacterales;Rhodobacteraceae;Sulfitobacter.
AF547424	AF468380	*Sulfitobacter*	97	FJ826092	61	96.9	Root;Bacteria;Proteobacteria;Alphaproteobacteria;Rhodobacterales;Rhodobacteraceae;Sulfitobacter.
AF547422	AY580666	Bacteroidetes	96	FJ745252	94	96.3	Root;Bacteria;Bacteroidetes;Flavobacteria;Flavobacteriales;Cryomorphaceae;unclassified_Cryomorphaceae.
AF547421	AB113609	β-Proteobacteria	93	DQ450189	208	94.4	Root;Bacteria;Proteobacteria;Betaproteobacteria;Burkholderiales;Comamonadaceae;unclassified_Comamonadaceae.

AF547415	AY345438	*Roseobacter*	99	EF574092	341	99.8	Root;Bacteria;Proteobacteria;Alphaproteobacteria;Rhodobacterales;Rhodobacteraceae;Loktanella.
AF547414	AJ459874	α-Proteobacteria	95	DQ493433	330	99.2	Root;Bacteria;Proteobacteria;Alphaproteobacteria;Caulobacterales;Caulobacteraceae;unclassified_Caulobacteraceae.
AF547413	AY424898	*Oceanicola*	97	FJ869046	341	99.8	Root;Bacteria;Proteobacteria;Alphaproteobacteria;Rhodobacterales;Rhodobacteraceae;unclassified_Rhodobacteraceae.
AF547409	AY580664	Bacteroidetes	94	FJ745252	97	95.6	Root;Bacteria;Bacteroidetes;Flavobacteria;Flavobacteriales;Cryomorphaceae;unclassified_Cryomorphaceae.
AF547408	AY580726	Bacteroidetes	92	AM279196	101	93.1	Root;Bacteria;Bacteroidetes;unclassified_Bacteroidetes.
AF547407	AY345438	*Roseobacter*	98	EF574092	341	99.3	Root;Bacteria;Proteobacteria;Alphaproteobacteria;Rhodobacterales;Rhodobacteraceae;Loktanella.
AF547406	AY038571	*Roseobacter*	97	DQ234154	61	99.5	Root;Bacteria;Proteobacteria;Alphaproteobacteria;Rhodobacterales;Rhodobacteraceae;unclassified_Rhodobacteraceae.
AF547405	AB114422	*Roseobacter*	97	AB114422	341	97.8	Root;Bacteria;Proteobacteria;Alphaproteobacteria;Rhodobacterales;Rhodobacteraceae;Roseovarius.
AF547399	AY580445	*Roseobacter*	99	FJ745263	61	98.9	Root;Bacteria;Proteobacteria;Alphaproteobacteria;Rhodobacterales;Rhodobacteraceae;Roseovarius.

References

González JM, Simó R, Massana R, Covert JS, Casamayor EO, Pedrós-Alió C, Moran MA: **Bacterial community structure associated with a dimethylsulfoniopropionate-producing North Atlantic algal bloom.** *Appl Environ Microbiol* 2000, **66**:4237-4246.

Hagström Å, Pommier T, Rohwer F, Simu K, Stolte W, Svensson D, Zweifel UL: **Use of 16S ribosomal DNA for delineation of marine bacterioplankton species.** *Appl Environ Microbiol* 2002, **68**:3628-3633.

Mou X, Moran MA, Stepanauskas R, González JM, Hodson RE: **Flow-cytometric cell sorting and subsequent molecular analyses for culture-independent identification of bacterioplankton involved in dimethylsulfoniopropionate transformations.** *Appl Environ Microbiol* 2005, **71**:1405-1416.

Pinhassi J, Simó R, González JM, Vila M, Alonso-Sáez L, Kiene RP, Moran MA, Pedrós-Alió C: **Dimethylsulfoniopropionate turnover is linked to the composition and dynamics of the bacterioplankton assemblage during a microcosm phytoplankton bloom.** *Appl Environ Microbiol* 2005, **71**:7650-7660.

Rusch DB, Halpern AL, Sutton G, Heidelberg KB, Williamson S, Yooseph S, Wu D, Eisen JA, Hoffman JM, Remington K, Beeson K, Tran B, Smith H, Baden-Tillson H, Stewart C, Thorpe J, Freeman J, Andrews-Pfannkoch C, Venter JE, Li K, Kravitz S, Heidelberg JF, Utterback T, Rogers YH, Falcón LI, Souza V, Bonilla-Rosso G, Eguiarte LE, Karl DM, Sathyendranath S, Platt T, Bermingham E, Gallardo V, Tamayo-Castillo G, Ferrari MR, Strausberg RL, Nealson K, Friedman R, Frazier M, Venter JC: **The Sorcerer II Global Ocean Sampling expedition: northwest Atlantic through eastern tropical Pacific.** *PLoS Biol* 2007, **5**:398-431.

Wang Q, Garrity GM, Tiedje JM, Cole JR: **Naïve Bayesian classifier for rapid assignment of rRNA sequences into the new bacterial taxonomy.** *Appl Environ Microbiol* 2007, **73**:5261-5267.

Yarza P, Richter M, Peplies J, Euzeby J, Amann R, Schleifer K-H, Ludwig W, Glöckner FO, Rosselló-Mora R: **The all-species living tree project: a 16S rRNA-based phylogenetic tree of all sequenced type strains.** *Syst Appl Microbiol* 2008, **31**:241-250.
